# Effects of Midazolam on the Development of Adult Leydig Cells From Stem Cells *In Vitro*

**DOI:** 10.3389/fendo.2021.765251

**Published:** 2021-11-12

**Authors:** Xingyi Zhao, Minpeng Ji, Xin Wen, Dan Chen, Fu Huang, Xiaoju Guan, Jing Tian, Jiajia Xie, Jingjing Shao, Jiexia Wang, Luoqi Huang, Han Lin, Leping Ye, Haolin Chen

**Affiliations:** ^1^ Zhejiang Provincial Key Laboratory of Anesthesiology, Department of Anesthesiology, The Second Affiliated Hospital and Yuying Children’s Hospital of Wenzhou Medical University, Wenzhou, China; ^2^ Department of Gynecology and Obstetrics, The Second Affiliated Hospital and Yuying Children’s Hospital of Wenzhou Medical University, Wenzhou, China; ^3^ Department of Pharmacology, The Second Affiliated Hospital and Yuying Children’s Hospital of Wenzhou Medical University, Wenzhou, China; ^4^ Department of Pediatrics, Peking University First Hospital, Beijing, China

**Keywords:** midazolam, stem Leydig cell, testosterone, progesterone, steroidogenesis, AKT-CREB signaling

## Abstract

**Background:**

Midazolam is a neurological drug with diverse functions, including sedation, hypnosis, decreased anxiety, anterograde amnesia, brain-mediated muscle relaxation, and anticonvulsant activity. Since it is frequently used in children and adolescents for extended periods of time, there is a risk that it may affect their pubertal development. Here, we report a potential effect of the drug on the development of Leydig cells (LCs), the testosterone (T)-producing cells in the testis.

**Methods:**

Stem LCs (SLCs), isolated from adult rat testes by a magnetic-activated cell sorting technique, were induced to differentiate into LCs *in vitro* for 3 weeks. Midazolam (0.1–30 μM) was added to the culture medium, and the effects on LC development were assayed.

**Results:**

Midazolam has dose-dependent effects on SLC differentiation. At low concentrations (0.1–5 μM), the drug can mildly increase SLC differentiation (increased T production), while at higher concentrations (15–30 μM), it inhibits LC development (decreased T production). T increases at lower levels may be due to upregulations of scavenger receptor class b Member 1 (SCARB1) and cytochrome P450 17A1 (CYP17A1), while T reductions at higher levels of midazolam could be due to changes in multiple steroidogenic proteins. The uneven changes in steroidogenic pathway proteins, especially reductions in CYP17A1 at high midazolam levels, also result in an accumulation of progesterone. In addition to changes in T, increases in progesterone could have additional impacts on male reproduction. The loss in steroidogenic proteins at high midazolam levels may be mediated in part by the inactivation of protein kinase B/cAMP response element-binding protein (AKT/CREB) signaling pathway.

**Conclusion:**

Midazolam has the potential to affect adult Leydig cell (ALC) development at concentrations comparable with the blood serum levels in human patients. Further studies are needed to test the effects on human cells.

## Introduction

Male infertility has been rising in recent decades, but the underlying cause is still unclear. A recent meta-regression study has found that during the last 50 years, the mean sperm counts of men declined 1.4% per year on average, with an overall decline of 52.4% between 1973 and 2011 (1). Testicular Leydig cells (LCs) are the main source of testosterone (T) in mammalian males, including men. T is critical for maintaining male reproductive functions, including spermatogenesis, and normal physiological functions (2, 3). Defects in LCs could result in low serum T level that might affect, in addition to reproduction, the general physiology of males, with symptoms including changes in body composition, increased fatigue, sexual dysfunction, depressed mood, decreased cognitive function, and reduced immune response (4–7). Thus, the development and maintenance of an appropriate functional LC population is of fundamental importance.

LC T production is a multistep process that involves mobilization of cholesterol and its conversion into T (8, 9). Two major mechanisms are used to provide cholesterol for steroidogenesis, *de novo* synthesis and import from the circulation by scavenger receptor class b Member 1 (SCARB1) (10). SCARB1 functions as a receptor for high-density lipoproteins, which carries cholesterol through the circulation (11). Within LCs, cholesterol can be stored in lipid droplets and the plasma membrane. Under stimulations by luteinizing hormone (LH) or other acute steroidogenic regulatory factors, cholesterol can be mobilized from the above locations and delivered into the inner mitochondrial membrane by steroidogenic acute regulatory protein (STAR) and translocator protein (TSPO) (12). Upon reaching the inner mitochondrial membrane, cholesterol is converted to pregnenolone by cytochrome P450 family 11A1 (CYP11A1). Pregnenolone then moves out of the mitochondria and reaches the smooth endoplasmic reticulum, where it is further metabolized into progesterone (P4), 17-hydroxy-P4, androstenedione, and T by a series of steroidogenic enzymes, including 3β-hydroxysteroid dehydrogenase (HSD3B), cytochrome P450 family 17A1 (CYP17A1), and 17β-hydroxysteroid dehydrogenase (HSD17B) (8, 9). The translocation of cholesterol across the mitochondrial membrane and its conversion to pregnenolone by CYP11A1 are considered the rate-limiting steps in steroidogenesis.

It is well-known that LC development and function are sensitive to various external influences, including nutrition, environmental contaminants, and drugs (9, 13, 14). Due to its unique properties for sedation, hypnosis, antianxiety, anterograde amnesia, and central muscle relaxation, and as an anticonvulsant, midazolam is among the top choices for managing sedation and epilepsy in children and adolescents (15–20). Since the drug can be used repeatedly for an extended period of time, its potential negative effects on neural development has been previously evaluated (21–24). It has been found that midazolam usage during puberty may affect brain development (25, 26). The pubertal period is also important for the development of other organs, including the reproductive system, which is particularly vulnerable to external factors during the period (27). Studies have shown that midazolam can acutely affect T secretion of adult LCs (28). The potential long-term effects of midazolam on LC development during puberty are still unknown.

Studies have shown that as in humans, LCs in rats are developed from the testicular interstitial stem LCs (SLCs) during puberty (29–31). In adult rats, LCs can be completely eliminated by a single dose of ethane dimethane sulfonate (EDS), after which a new generation of LCs can be reestablished from SLCs within 2 months. The regeneration process and the stem cells involved are the same as those in puberty (32–35). SLCs have been found to express multiple protein markers, including nestin, nuclear receptor subfamily 2F2 (NR2F2), Aristaless-related homeobox gene (ARX), cluster of differentiation 51 (CD51), p75 neurotropin receptor (p75NTR), platelet-derived growth factor receptor alpha (PDGFRA), transcription factor 21 (TCF21), glioma-associated oncogene homolog 1 (GLI1), endosialin, and cluster of differentiation 90 (CD90) (36–38). These markers were identified by various groups with different species, with few being tested in rats. Also, most of these markers are not membrane-bound, so they cannot be used for SLC isolation by routine methods. Two of these membrane-associated markers (PDGFRA and CD90) were tested in rats and successfully used for SLC isolation in that species (39–41).

The SLCs isolated by these markers can expand *in vitro* and differentiate into LCs with relatively simple medium. For example, CD90-positive SLCs can be differentiated with high efficiency by a very simple medium that contains only LH and a desert hedgehog (DHH) agonist (39, 41). CD90 appears to be more specific for SLCs than PDGFRA (41). In the present study, we isolated CD90+ SLCs by a magnetic-activated cell sorting (MACS) technique and used the cells to test potential effects of midazolam on SLC proliferation and differentiation. Our results show that with concentrations that are comparable with the blood serum levels in human patients (78–1,103 ng/ml, equivalent to 0.25–3.5 μM) (42–44), midazolam can significantly affect SLC proliferation and differentiation.

## Materials and Methods

### Chemicals and Reagents

DMEM/F12 medium, dexamethasone, fetal bovine serum (FBS), insulin/transferrin/selenium (ITS), and bovine serum albumin (BSA) were purchased from Sigma-Aldrich (St. Louis, MO). β-Mercaptoethanol, fibroblast growth factor 2 (FGF2), N2, and B27 supplements were from Thermo Fisher (Waltham, MA). Chicken embryo extract was from Biological (Salem, MA). Epidermal growth factor (EGF) was from PeproTech (Rocky Hill, NJ). Leukemia inhibitory factor (LIF) was from Millipore (Burlington, MA). Oncostatin-M and platelet-derived growth factor bb (PDGFBB) were from ProSpec (East Brunswick, NJ). Smoothened agonist (SAG) was purchased from Cayman Chemical (Ann Arbor, MI). Anti-R-phycoerythrin (PE) Magnetic Particles and BD IMag™ Buffer (10×) were from BD Biosciences (Franklin Lakes, NJ). Midazolam (stock solution: 5 mg/ml in aqueous solution) was from Jiangsu Enhua Pharmaceutical (Xuzhou, China). Human LH was from MyBioSource (San Diego, CA). Detailed information for other materials can be found in **Supplementary Table S1**. The manufacturers and the dilutions of the antibodies can be found in **Supplementary Table S2**. The primers for QPCR are summarized in **Supplementary Table S3**.

### Animals and Treatments

Adult male Sprague Dawley rats, 2–4 months of age, were purchased from Shanghai Laboratory Animal Centre (Shanghai, China). The rats were housed in the animal maintenance facility of Wenzhou Medical University under controlled light (12-h light:12-h dark) and temperature (22°C) with free access to water and rat chow. All experimental procedures were approved by the Institutional Animal Care and Use Committee of Wenzhou Medical University and were in accordance with the NIH Guide for the Care and Use of Laboratory Animals. After 1-week adjustment, rats were injected with a single intraperitoneal dose of EDS (i.p., 80 mg/kg of BW) dissolved in a mixture of dimethyl sulfoxide (DMSO):PBS (1:3) to eliminate the LC population as previously described (45). SLCs were purified from the testes of rats 4 days after EDS treatment, by which time all LCs had been eliminated from the testes.

### Magnetic-Activated Cell Sorting for Purification of Stem Leydig Cells From Rat Testes

Testes were collected from rats after their euthanasia by CO_2_ exposure. After decapsulation, the testicular parenchyma was digested in DMEM/F12 medium containing 1 mg/ml of collagenase IV at 34°C for 30 min with slow shaking (90 cycles/min). After settling for 1 min, the supernatants were filtered through 70-μm pore nylon mesh. Positive selection of CD90+ cells was carried out by the MACS protocol according to the instructions of the manufacturer (BD IMag™). The cell pellets were suspended in cold BD IMag™ (BI) buffer at a density of 2 × 10^7^ cells/ml and stained with PE-conjugated CD90 antibodies (1:500) at 4°C in darkness for 40 min. Cells were then washed twice with BI buffer and labeled with anti-R-PE Magnetic Particles (1:100) for 30 min at 4°C. The magnetic particle-labeled cells were transferred to a collection tube, which was immediately placed onto the BD IMag™ Cell Separation Magnet hold (BD Biosciences, USA). After being incubated for 8 min, the supernatant regarded as a negative fraction was collected, while the positive fractions that adhered to the wall of the tube was resuspended with BI buffer. The sorting procedure was repeated twice more with the positive fraction to further purify the positive cells. The final positive cell fraction was suspended in PBS, and the percentage of CD90+ cells was determined by flow cytometer, or expanded *in vitro* for 7 days.

### Culture and Treatments of the Purified Stem Leydig Cells

The purified SLCs were seeded in a 24-well plate with DMEM/F12 medium containing 10% FBS. After the SLCs were attached to the bottom of the well for 3 h, the medium was replaced by an expansion medium (EM) as described previously (41, 46). The EM was formulated with DMEM/F12 and the following components: BSA (0.1%), dexamethasone (0.5 nM), ITS (1×), LIF (0.5 ng/ml), chicken embryo extract (2.5%), β-mercaptoethanol (50 μM), non-essential amino acids (0.5%), N2 supplements (0.5%), B27 supplements (1%), FGF2 (10 ng/ml), EGF (10 ng/ml), PDGFBB (10 ng/ml), oncostatin-M (10 ng/ml), and FBS (2.5%). Cells were cultured at 34°C with 5% CO_2_ for 1 week. After expansion, cells were stained for PE-CD90 antibodies to have their purities checked by a flow cytometer, collected for QPCR analysis of testicular-related genes, or switched to the differentiation inducing medium (DIM) for 3 weeks (41). DIM was made from DEME/F12 that contained LH (10 ng/ml) and SAG (0.5 μM). After 3 weeks, the medium was collected for T and P4 assay.

To examine the effects of midazolam on SLC proliferation, cells were incubated with different concentrations of midazolam (0.1–30 μM; <0.2% solvent from the stock) during the last 48 h of the expansion period. Cells were then labeled with ethynyl-2′-deoxyuridine (EdU) in the last 24 h of midazolam treatment. To examine the effects of midazolam on SLC differentiation, SLCs were incubated with different concentrations of midazolam (0.1–30 μM) in DIM for 21 days after the expansion period. The medium, which was changed every 3 or 4 days during differentiation, was collected for T and P4 assay. By the end of 3 weeks, the cells were fixed and stained for CYP11A1 or collected for QPCR or Western blotting analysis.

### Measurement of Cell Viability (MTS Assay)

SLCs obtained by MACS were seeded into a 96-well plate and expanded for 7 days. During the last 24 h, the cells were exposed to midazolam (0–30 μM). CellTiter 96^®^ Aqueous One Solution (Promega, USA) was used to detect cell viability according to the manufacturer’s instructions. In brief, during the last 3 h of the 24-h midazolam exposure, 20 μl of CellTiter 96 reagent was added into each well of 200 μl medium, and cells were incubated at 37°C for 3 h. Thereafter, the cell viability was measured by detecting the absorbance at 490 nm using BioTek Microplate Reader (BioTek, USA).

### Immunofluorescence Staining

For EdU or CYP11A1 immunofluorescence staining, SLCs were plated in a 24-well plate containing a TC-treated round cover slide (WHB Scientific, Shanghai, China; Cat#, WHB-24-CS). Cells were expanded and differentiated by a routine procedure. SLC proliferation was determined by the Click-it^®^ EdU Alexa Fluor Kit (Invitrogen, USA) according to the manufacturer’s instructions. In brief, the EdU-labeled cells were fixed with 4% paraformaldehyde for 30 min at room temperature followed by breaking the membrane with 0.5% Trion X-100. After being washed with PBS, the cells were treated with Click-it reagents to reveal nuclear EdU incorporation, followed by 4′,6-diamidino-2-phenylindole dihydrochloride (DAPI) staining of the nucleus. For CYP11A1 staining, the cells exposed to midazolam (5 and 30 μM) for 21 days were first fixed with 4% paraformaldehyde for 30 min, followed by incubation with primary antibodies (1:100) at 4°C overnight in darkness. After being washed with PBS, the cells were stained with fluorescent secondary antibodies for 1 h, followed by DAPI staining of the nucleus. The EdU+ cells and total nuclei stained by DAPI were counted under a ×20 objective.

### Flow Cytometry Analysis of CD90-Positive Cells

The percentages of CD90+ cells were analyzed in the freshly prepared testicular cell suspensions before or after PE-CD90 antibody staining, after sorting by MACS, or after expansion of the cells *in vitro* for 1 week. Cultured cells were released from the culture plate by trypsin-EDTA and washed twice with PBS. The cells were then suspended in PBS and stained with PE-CD90 antibody for 45 min. After being washed three times with PBS, cells were analyzed with an Attune™ NxT Flow Cytometer (Thermo Fisher, USA). The parameter setting and background signal correction were done as previously published (41).

### Ultra Performance–MS/MS Assay of Testosterone and Progesterone

T and P4 in the media were assayed with an Acquity ultra performance liquid chromatography unit and XEVO TQD triple quadrupole mass spectrometer (Waters Corp., USA), according to previous protocols (47, 48), with minor modification. In brief, working solutions of both T/P4 were diluted from stock solutions with methanol. Calibration standards and quality control samples were prepared with T/P4-free cell culture media. The internal standard (IS) working solution was made from the T-d3 stock solutions. The IS working solution (10 μl) was added to the collected cell medium (100 μl) and mixed with 200 μl of acetonitrile. Then the mixture was centrifuged at 12,000*g* for 15 min. The supernatant (10 μl) was loaded into the machine by an auto-sampler. The acceptance of validation tests for intra-assay and inter-assay variabilities was selected at ≤ ± 15% for both T and P4.

### RNA Extraction and Quantitative PCR

Total RNA was extracted with RNeasy^®^ Mini Kit (Qiagen, Germany) according to the manufacturer’s instructions. cDNA and QPCR analyses were performed as previously reported (49). We analyzed the mRNA levels of testicular cell marker genes of LCs (*Lhcgr*, *Star*, *Cyp11a1*, *Cyp17a1*, *Hsd3b1*, and *Insl3*), SLCs (*Gli1*, *Cd90*, and *Pdgfra*), immune cells (*Ptprc*), macrophages (*Adgre1*), smooth muscle cells (*Acta2*), germ cells (*Smc1b* and *Ddx4*), vascular endothelial cells (*Vwf*), and Sertoli cells (*Sox9*). The mRNA levels of ribosomal protein S16 (*Rps16*) were used as internal controls, and the Ct value of target genes was normalized to that of *Rps16* as described (49).

### Western Blotting

Western blotting was done according to a protocol reported previously (50). Briefly, total cellular protein was extracted by radioimmunoprecipitation assay (RIPA) Lysis Buffer (Beyotime, China), and the protein concentrations were measured by Enhanced BCA Protein Assay Kit (Beyotime, China). Sample proteins (30 μg) were separated by 10% sodium dodecyl sulfate–polyacrylamide gel electrophoresis (SDS-PAGE) and then transferred to a polyvinylidene fluoride membrane. To save samples and antibodies, the membranes were then cut into multiple strips according to the target protein molecular weights (**Supplementary Table S4**). The antibodies used include SCARB1, LHCGR, STAR, CYP11A1, HSD3B1, CYP17A1, INSL3, AKT, pAKT, CREB, pCREB, and α-tubulin. The density of each target protein band that had a correct molecular weight was quantified using Image Lab 3.0 (Bio-Rad, USA) and adjusted to IS α-tubulin. The manufacturers and the dilutions of the antibodies are listed in **Supplementary Table S2**.

### Statistical Analysis

Data are expressed as means ± SEM. One-way ANOVA was used for multiple group comparisons. Significant differences among individual groups were determined by the Student–Newman–Keuls (SNK) test, using the SPSS statistical software package. GraphPad Prism 8.0.1 was used to draw diagrams. Differences were considered significant at p < 0.05.

## Results

### Purification of CD90+ Stem Leydig Cells by Magnetic-Activated Cell Sorting and Expansion Procedure

**Figure 1:**
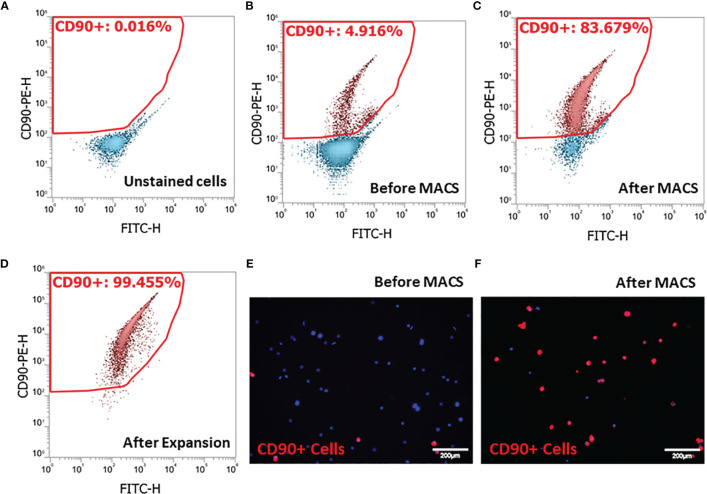
Isolation of stem Leydig cells by the surface marker CD90: **(A)** unstained testicular cell suspension. **(B)** Testicular cell suspension stained with PE-CD90 antibody before isolation by magnetic-activated cell sorting (MACS). **(C)** PE-CD90 antibody-stained cells after MACS. **(D)** CD90+ cells after their culture *in vitro* for 7 days. **(E)** CD90+ cells before MACS. **(F)** CD90+ cells after MACS.

Since SLCs in the rat testis specifically express the surface marker CD90 (39, 41), we adopted an SLC isolation procedure based on MACS protocol. Cell suspensions prepared by collagenase digestion of EDS treated rat testes were analyzed by flow cytometry before (**Figure 1A**) or after PE-CD90 antibody staining (**Figures 1B–D**). Since testicular cells have high autofluorescence, it is important to correct the background signals to avoid false-positive cells. Usually, the autofluorescence is not wavelength-specific and can emit light across multiple channels with similar intensity. Consequently, we used fluorescein isothiocyanate (FITC) channel (x-axis) readings to correct possible background interference signals in PE channel (y-axis). With FITC and PE channels set as x- and y-axes, the cells with autofluorescence distribute along the diagonal line (**Figure 1A**). Cells with specific PE-CD90 antibody staining could appear on the top of the diagonal line (4.9%, **Figure 1B**). The PE-CD90 antibody-stained cells were positively selected with a MACS system (BD Imag™). The selection protocol enriched CD90+ cells from an initial 4.9% purity (**Figures 1B, E**) to about 83% (**Figures 1C, F**). However, after a period of 7 days of *in vitro* expansion, the purity of CD90+ cells reached almost 100% (**Figure 1D**).

**Figure 2:**
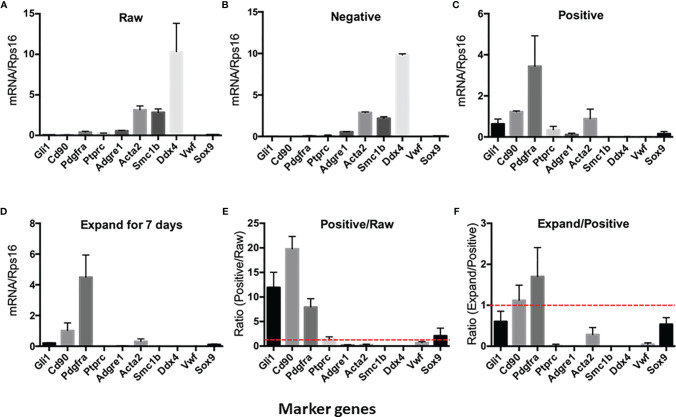
Expression of testicular cell markers by cell suspensions at different enrichment steps: **(A)** Raw testicular cells before magnetic-activated cell sorting (MACS). **(B)** CD90− cells after MACS. **(C)** CD90+ cells after MACS. **(D)** CD90+ cells cultured in expansion medium for 7 days. **(E)** Enrichment factors for each cell type by MACS procedure (ratios of CD90+ cells and raw cells). **(F)** Enrichment factors for each cell type by *in vitro* expansion (ratios of expanded cells and freshly sorted CD90+ cells). A ratio above onefold (broken red line) indicates positive enrichment. Data are expressed as mean ± SEM of four experiments.

To further characterize the purity of CD90+ cells, we have isolated the total RNAs from the cell suspensions and ran QPCR against the marker genes for each of the major testicular cell types (**Figure 2**). The cells and the corresponding markers include the following: SLCs (*Gli1*, *Cd90*, and *Pdgfra*), immune cells (*Ptprc*), macrophages (*Adgre1*), smooth muscle cells (*Acta2*), germ cells (*Smc1b* and *Ddx4*), vascular endothelial cells (*Vwf*), and Sertoli cells (*Sox9*). The major cell types in the raw- and negative-cell suspensions after selection were germ cells, smooth muscle cells, and macrophages (**Figures 2A, B**). The positive cells enriched after MACS procedure expressed high levels of all three SLC markers (*Gli1*, *Cd90*, and *Pdgfra*) and smooth muscle cell markers (*Acta2*) (**Figure 2C**). Interestingly, after 7 days of *in vitro* expansion, the high expressions of all three SLC genes were maintained, while the markers of other cells decreased significantly (**Figure 2D**). The enrichment factors (ratios) were calculated for each cell type by dividing the expression levels of the marker genes before and after a particular procedure (**Figures 2E, F**). The values above the red line (ratio of 1) indicate positive enrichment by the procedure. Clearly, the MACS procedure positively enriched SLCs (**Figure 2E**), while the *in vitro* expansion step negatively enriched all other cell types except SLCs (**Figure 2F**).

### Effects of Midazolam on Stem Leydig Cell Viability and Proliferation

**Figure 3:**
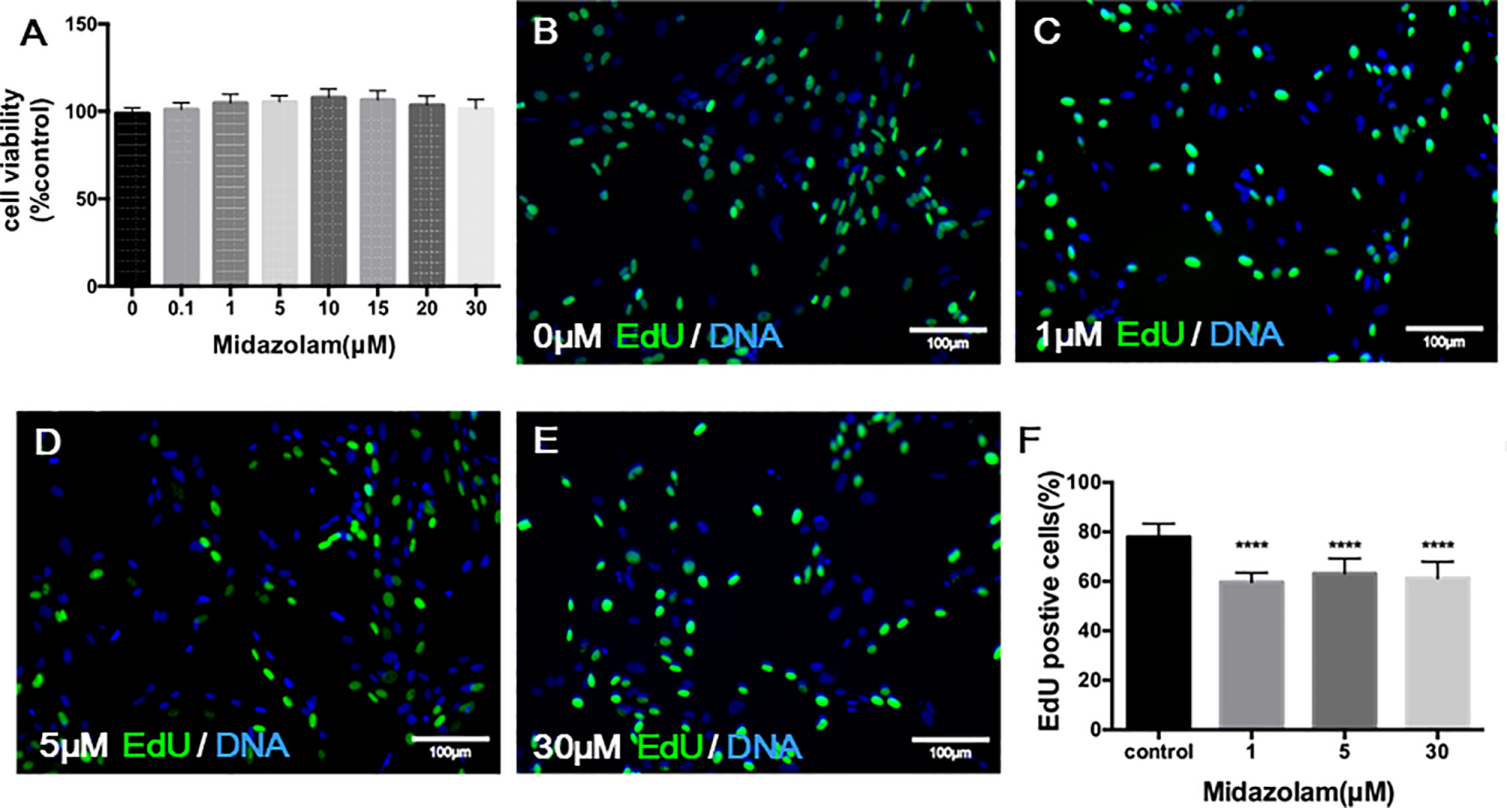
Effects of midazolam on the viability **(A)** and proliferation **(B–F)** of stem Leydig cells (SLCs): **(A)** SLCs were incubated with midazolam for 24 h by the end of the expanding period, and a tetrazolium compound was added to the culture during the last 3 h. The formazan product formed by living cells was qualified by absorbance at 490 nm. **(B–F)** SLCs were incubated with different concentrations of midazolam **(B)**, 0 μM; **(C)**, 1 μM; **(D)**, 5 μM; and **(E)**, 30 μM) for 48 h by the end of the expanding period; during the last 24 h, the cells were labelled by EdU. Nuclei of the dividing cells were stained in green, and all cell nuclei were stained in blue (4′,6-diamidino-2-phenylindole dihydrochloride (DAPI)). **(F)** Quantification of EdU-labeled cells as % of total cells. Data were expressed as mean ± SEM of four experiments. Significantly different from the control group at ^****^p < 0.0001.

To examine the potential toxic effect of midazolam on SLC viability, we have incubated cells with different concentrations of midazolam for 24 h at the end of the expansion period. The ability of cells to convert tetrazolium into a formazan product within 3 h, which reflects cell viability, did not change across all midazolam concentrations, ruling out a general toxic effect on the cells (**Figure 3A**). To examine the effects of midazolam on SLC proliferation, the CD90+ cells were cultured with different concentrations of the drug for 48 h at the end of the expansion period, with EdU added during the last 24 h. The results showed that midazolam resulted in mild (about 20%) inhibition of cell proliferation (**Figures 3B–F**). Interestingly, the inhibitory effect occurred at concentrations as low as 1 μM without further reductions with concentrations up to 30 μM.

### Effect of Midazolam on Stem Leydig Cell Differentiation

**Figure 4:**
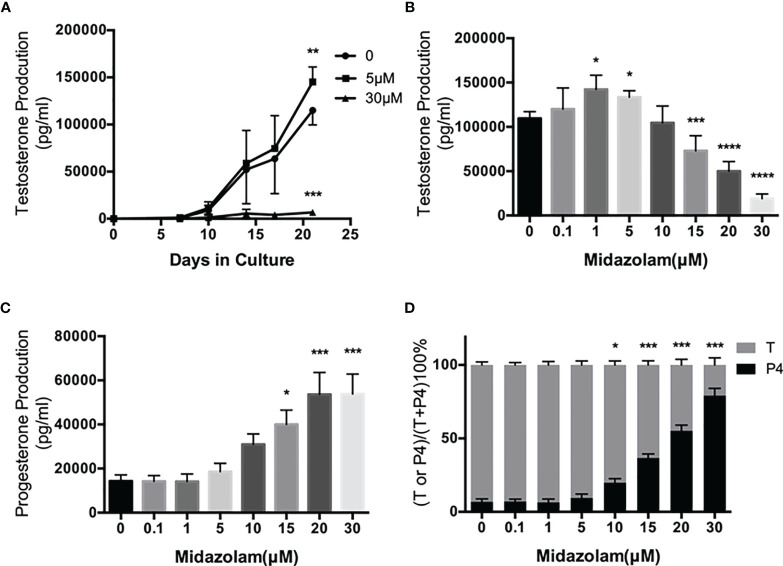
Effects of midazolam on differentiation of stem Leydig cells (SLCS): **(A)** SLCs were induced to differentiate into testosterone-producing Leydig cells in 21 days in the presence of 5 or 30 μM of midazolam. Testosterone (T) concentrations were assayed in the culture medium collected every 3–4 days. **(B)** T concentrations in the day 21 culture media. **(C)** Progesterone (P4) concentrations in the day 21 culture media. **(D)** Percentages of T and P4 in the day 21 culture media. Data are expressed as mean ± SEM of four to six experiments. The experimental repeats for each figure are as follows: **(A)** (4), **(B)** (6), **(C)** (4), and **(D)** (4) . Significantly different from the control groups at ^*^p < 0.05, ^**^0.01, ^***^0.001, or ^****^0.0001.

To examine the effects of midazolam on SLC differentiation, cells were induced to differentiate into adult LCs (ALCs) *in vitro* for 3 weeks (**Figure 4**). Without midazolam, SLCs, which initially were steroidogenically inactive, became T-producing cells by 10 days (**Figure 4A**). The ability to produce T kept increasing through 21 days. With a low level (5 μM) of midazolam, there was a mild, but significant, increase in T production by day 21. However, with higher midazolam concentration (30 μM), T production was almost completely inhibited through day 21 (**Figure 4A**). The biphasic responses to midazolam were confirmed by incubating cells with increasing concentration of the drug during the 21-day differentiation process (**Figure 4B**). T production was upregulated by low levels (1–5 μM) and downregulated by higher concentrations (15–30 μM).

During steroidogenesis, P4 is made as an intermediate precursor for T. We have assayed P4 in the culture medium (**Figure 4C**). To our surprise, P4 concentrations were upregulated by midazolam concentrations above 15 μM, and consequently, the proportions of T decreased while P4 were increased at these concentrations (**Figure 4D**).

### Effect of Midazolam on Steroidogenic Enzymes

**Figure 5:**
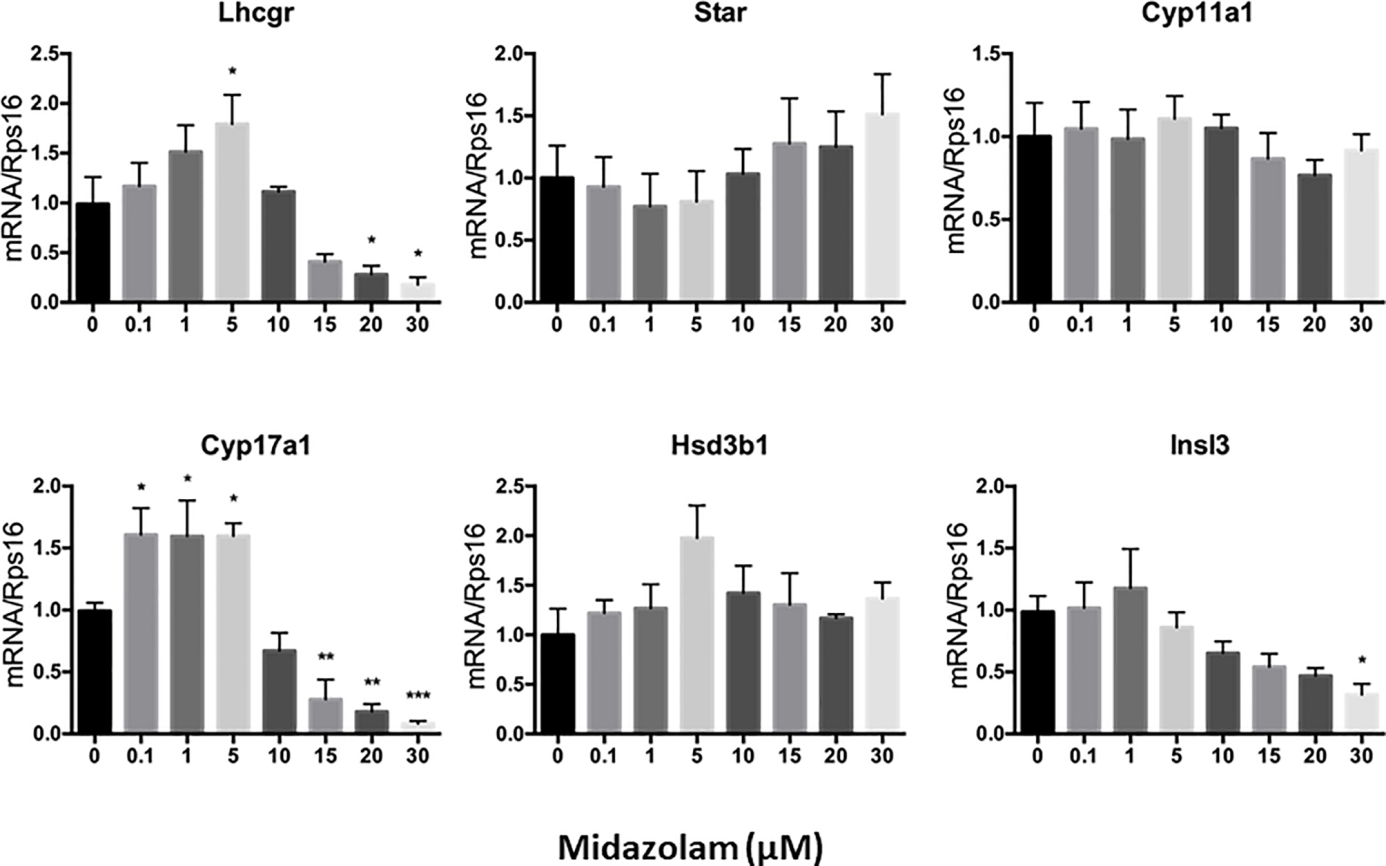
Effects of midazolam on the expression of steroidogenic genes: stem Leydig cells were induced to differentiate into Leydig cells in the presence of different concentrations of midazolam for 21 days. Expressions of steroidogenic genes were assayed by QPCR with *Rps16* as an internal control. Data are expressed as mean ± SEM of five experiments. Significantly different from the control groups at ^*^p < 0.05, ^**^0.01, or ^***^0.001.

**Figure 6:**
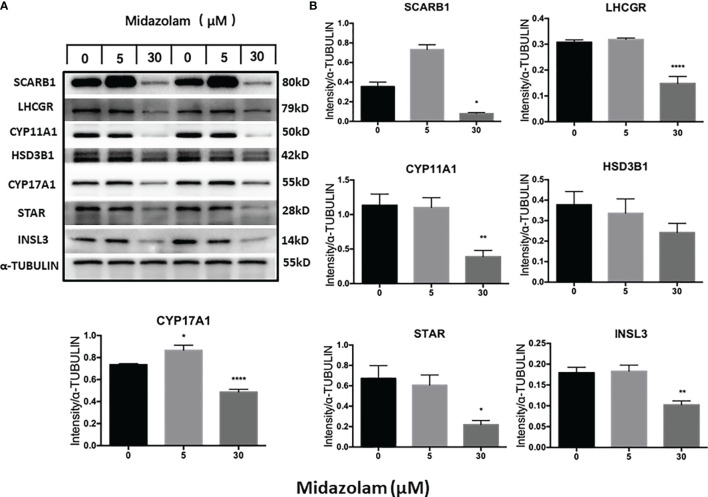
Effects of midazolam on the expression of steroidogenic proteins: stem Leydig cells were induced to differentiate into Leydig cells in the presence of 5 or 30 μM of midazolam for 21 days. Steroidogenic proteins were assayed by Western blotting with α-tubulin as an internal control. **(A)** Representative Western blotting bands for each protein of two individual experiments. **(B)** Quantification of the Western blotting data, which are expressed as mean ± SEM of six experiments. Significantly different from the control groups at ^*^p < 0.05, ^**^0.01, or ^****^0.001.

To elucidate the specific effects of midazolam on the steroidogenic pathway, the mRNA and protein levels of the key steroidogenic enzymes were analyzed by QPCR (**Figure 5**) and Western blotting (**Figure 6**). With increasing concentrations of midazolam, two genes, LH/choriogonadotropin receptor (*Lhcgr*) and *Cyp17a1*, were first upregulated and then downregulated (**Figure 5**), while another four genes were not significantly affected. These biphasic responses of *Lhcgr* and *Cyp17a1* are consistent with T productions (**Figure 4**). Interestingly, the Western blotting showed a slightly different story (**Figure 6**). First, the low level midazolam-dependent upregulation of *Lhcgr* was no longer seen, while scavenger receptor class b member 1 (SCARB1) and CYP17A1 being significantly upregulated at low midazolam level (5 μM). Second, the midazolam-dependent high-level downregulations were seen in all proteins except HSD3B1. These results indicate that the effects of midazolam on the steroidogenic pathway were not even across all the genes and proteins and that the effects might be at both the transcriptional and translational steps.

**Figure 7:**
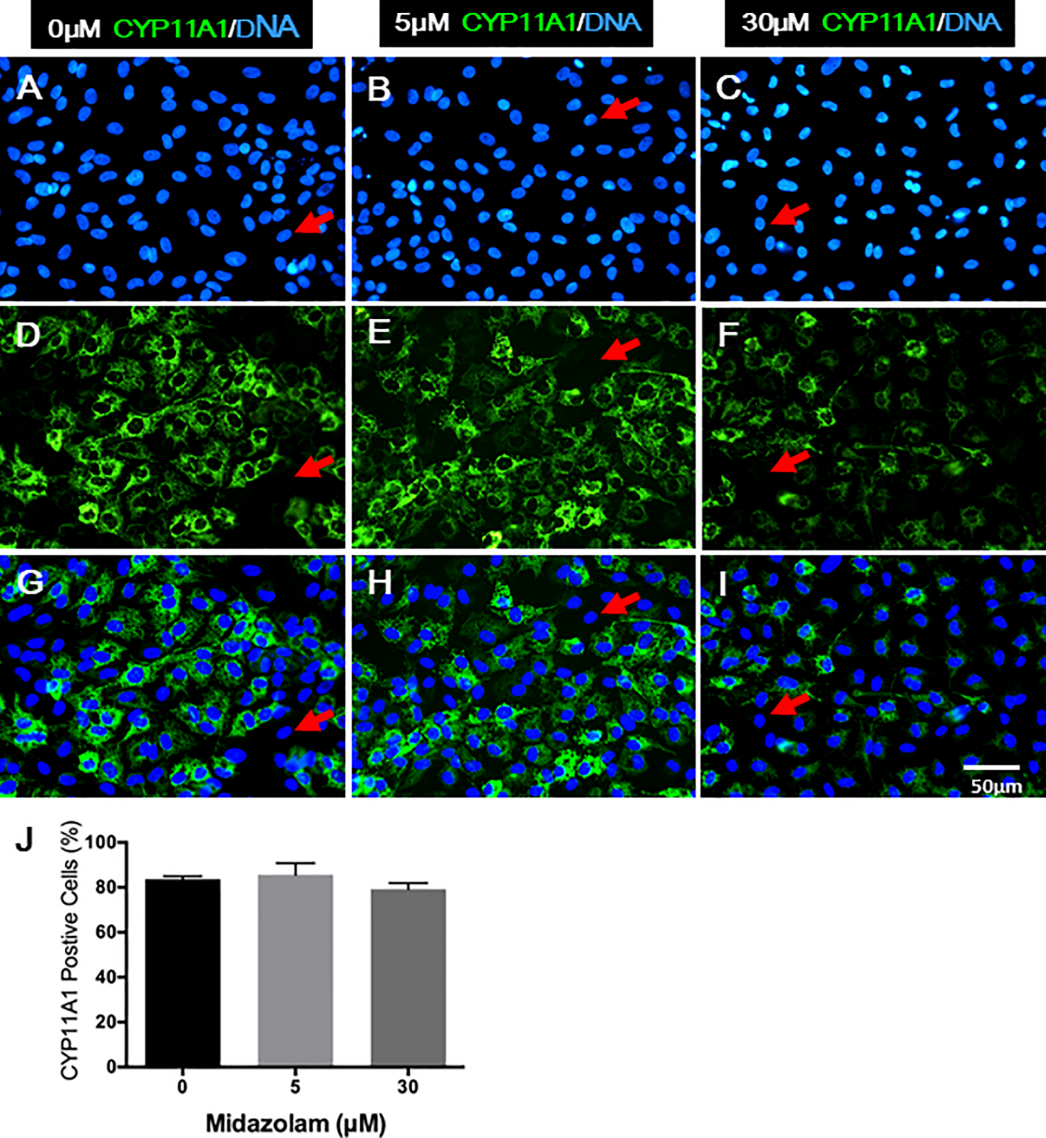
Effects of midazolam on the differentiation of CYP11A1+ cells: stem Leydig cells were induced to differentiate into Leydig cells in the presence of 5 or 30 μM of midazolam for 21 days. CYP11A1 was stained by immunofluorescence (green). Nuclei were stained by DAPI (blue). Control **(A, D, G)**; 5 μM of midazolam **(B, E, H)**; 30 μM of midazolam **(C, F, I)**. **(J)** Quantification of CYP11A1+ cells from three doses. Photos are representatives of three experiments with similar results. Red arrows: examples of negative cells.

Changes in T production and steroidogenic pathway proteins could result from either a low number of LCs differentiated or cells with different steroidogenic activity. To examine how midazolam may affect LC numbers, cells were stained for CYP11A1 expression by immunofluorescence (**Figure 7**). The results showed that without midazolam (0 μM), significant numbers of SLCs can be induced to differentiate into LCs by 21 days. With low-dose (5 μM) exposure, CYP11A1 expression did not change significantly, while with high-dose (30 μM) exposure, CYP11A1 expression was significantly reduced (**Figures 7A–I**). However, quantification of CYP11A1+ cells indicated that the number of CYP11A1+ cells did not change significantly among the three groups (**Figure 7J**), suggesting that high levels of midazolam may only affect the expression of CYP11A1, not the number of LCs formed.

### Effect of Midazolam on AKT-CREB Signaling Pathway

**Figure 8:**
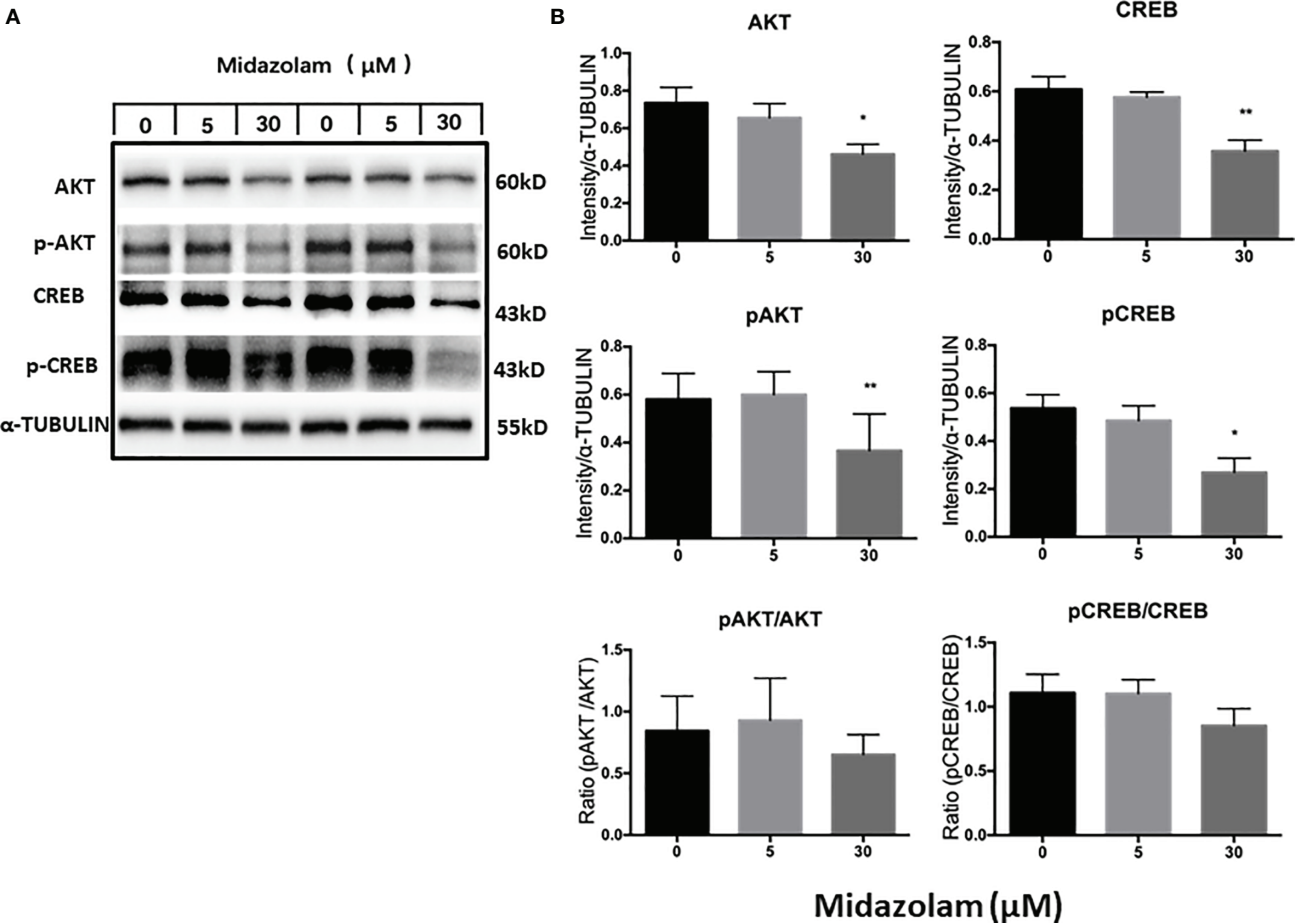
Effects of midazolam on the expressions of AKT-CREB signaling molecules. **(A)** Stem Leydig cells were induced to differentiate into Leydig cells in the presence of 5 or 30 μM of midazolam for 21 days. Expressions of AKT, CREB, and their phosphorylated forms were analyzed by Western blotting with α-tubulin as an internal control. These Western blotting bands were from two individual experiments. **(B)** Quantification of the Western blotting results. Data are expressed as mean ± SEM of three experiments. Significantly different from the control groups at ^*^p < 0.05 or ^**^0.01.

The PI3K/AKT signaling pathway has been shown to play an important role in steroidogenic cell development, function, and apoptosis (51–53). To examine whether this signaling pathway is affected by midazolam treatment, the protein expression levels and phosphorylation of the two key components in the pathway, AKT and CREB, were analyzed by Western blotting (**Figure 8**). The results showed clearly that the levels of the total and the phosphorylated forms of the both proteins were downregulated by high-level midazolam. However, the ratios of phosphorylation and total protein did not change significantly for the two proteins, suggesting that high-level midazolam may only affect the basal activities of the signaling molecules, not their acute responses. Also, no difference was detected for the two proteins at low dose, suggesting that this signaling pathway may be partially responsible for the reductions in T productions by high doses but was not responsible for the T increases in the low midazolam concentrations.

## Discussion

Testicular SLCs have been studied intensively during the past 10 years due to their potential use in cell-based therapies for hypogonadism. The majority of the *in vitro* studies of the cells used fluorescence-activated cell sorting (FACS) to isolate the cells (39, 41, 46, 54). This technique has many advantages for obtaining cells with high purity, but it also has limitations. FACS takes a relatively long time to sort cells, thus increasing chance of contamination. Also, cell viability can be affected by passage of the cells through a high pressured nozzle. Finally, FACS requires professional training. In order to overcome these limitations, we developed a new way of isolating SLCs by MACS protocol.

Our results indicated that the procedure can enrich the cells from 5% to about 83%. Interestingly, after 1-week *in vitro* expansion, the cell purity increased to almost 100%. The reason for this dramatic *in vitro* enrichment is still unclear. In addition to possible deaths of CD90− cells, it was most likely due to a cell diluting effect since SLCs expanded substantially during the week, while other cells may not divide or divide as quickly. The disagreement in the enrichment of three SLC markers (GLI1, PDGFRA, and CD90) also suggests the possibility that SLCs changed their gene expression properties during the *in vitro* expansion period. This is unlikely the reason the cell purity was increased since CD90 expression did not change significantly. Overall, the results indicated that MACS plus *in vitro* expansion could become an excellent protocol to purify SLCs from adult rat testes. The method is simple and fast and has the flexibility of scaling up or down to meet various experimental designs. More importantly, the method is less technically demanding and can be adapted to various lab settings, especially for those without access to a flow cytometer with sorting capacity.

With these high-quality SLCs on hand, we tested their application in a toxicology study. Midazolam is a benzodiazepine drug used widely by clinicians to manage neurological and mental disorders. Due to its diverse usage and potential to be used for extended periods of time in children and adolescents, there is a risk that the drug may affect the pubertal development of adolescents. In this study, we evaluated the effects of midazolam on LC development from its stem cells and found dose-dependent effects of the drug. Midazolam can either stimulate or inhibit SLC development, depending on the concentration. As previously reported, purified CD90+ SLCs can be induced to develop into T-producing LCs *in vitro* within 3 weeks in the presence of LH and SAG (a DHH agonist) (39, 41). The SLCs, which were initially steroidogenically inactive and did not express the major steroidogenic genes/proteins, became steroidogenically active and T-producing by about 10 days. The ability to produce T kept increasing through 3 weeks, indicating normal LC development *in vitro*. However, adding midazolam in the culture medium significantly affected the developmental process. At a low concentration range (1–5 μM), midazolam significantly increased ALC development and T production by the end of 3 weeks. However, at a higher concentration range (15–30 μM), midazolam significantly decreased T production, suggesting that the drug has a biphasic effect on ALC development, depending on its concentration.

T is synthesized from cholesterol by a multiple-enzymatic process, in which P4 is made as an intermediate product. To examine the mechanisms by which T production is specifically affected by different concentrations of midazolam, steroidogenic pathway genes/proteins were analyzed by QPCR and Western blotting. SCARB1 and CYP17A1 were upregulated by low midazolam concentrations that resulted in increased T and downregulated by higher concentrations by which T was downregulated. In addition, multiple other steroidogenic-related proteins, including LHCGR, STAR, and CYP11A1, were downregulated by high concentrations, but not affected by low concentrations, suggesting that SCARB1 and CYP17A1 may be the primary reason that T production was increased by low concentrations, while changes in multiple steroidogenic proteins may be responsible for the T reductions by higher concentrations.

An interesting observation is the dose-dependent increases in P4 production. It is well known that in addition to T, P4 may also affect male reproductive behavior and/or functions by acting on the brain (55, 56). The increases in P4 therefore may have physiological impact in the males. Another issue that needs to be discussed is the mechanism that is responsible for P4 upregulation. One might expect that P4 accumulation has to be caused by an increase in production, a decrease in metabolism, or a combination of the two. However, in rare circumstances, P4 may also accumulate if the reduction in its metabolism is more severe than the reduction in its production. The Western blotting results did not support any of these hypotheses since all the key enzymes, including STAR and CYP11A1, were downregulated. Steroidogenesis is a complex process that involves not only the steroidogenic enzymes themselves but also the membrane environments and co-factors involved, such as NADPH and NAD+. The regulation of steroidogenic enzymatic activities at the cell organelle level is not well-understood. Also, there could be changes in the ratio of delta-4 and delta-5 pathways, thereby shifting the accumulation of intermediate steroids. Clearly, additional studies are required to address the mechanism by which P4 increased while the total steroidogenic capacity was reduced.

Insulin-like 3 (INSL3) is a peptide hormone specifically produced by LCs in both fetal and adult testes (57). The hormone plays a critical role in the descent of fetal testes, but its role in the adult is still unclear. Recent studies suggest a role for INSL3 in extra gonadal physiology, including cardiovascular and bone health (58). Since INSL3 is not acutely regulated by the hypothalamic–pituitary axis, it is constitutively synthesized by LCs independent of steroidogenesis but nonetheless a functional marker for LCs (59). In the current study, INSL3 content was also reduced by the high concentrations of midazolam, suggesting that the inhibitory effects of the drug on LC development may not be restricted to the steroidogenic pathway.

It is well-known that LH plays a critical role in LC development and function. The finding that high concentrations of midazolam can reduce LHCGR expression suggests that some of the phenotypes observed under these conditions could result from LH under-stimulation. It has been reported that LH can stimulate LC STAR expression by activating the PKA-CREB pathway (60–62). In theca cells, a steroidogenic cell type in the ovary, LH regulates the expression of CYP17A1 and steroidogenesis through the AKT pathway (63, 64). In mouse LCs, AKT-CREB signaling was also found to be involved in the regulation of T production (65). In the current study, to verify the involvement of AKT-CREB signaling in midazolam effects, we assayed the levels of the total protein and phosphorylated forms of the two members by Western blotting. The results clearly showed that both total proteins and the phosphorylated forms were significantly reduced by high concentration of midazolam. However, the ratios of the phosphorylated forms and the total proteins did not change significantly, suggesting that the basal activity of the AKT-CREB signaling pathway, but not the acute regulations of the pathway, might be affected by high levels of midazolam. More work is needed to address the mechanisms that are responsible for the dose-dependent effects of midazolam on LC development.

Recent studies have found that various anesthetics might have the potential to affect LC functions directly or indirectly (50, 66–68). However, most of these studies focused on acute effects on well-developed adult LCs, not on the development of the cells. Midazolam is a benzodiazepine drug that is able to bind peripheral benzodiazepine receptor (PBR). PBR, also known as TSPO, is expressed at a high level in LCs (69). Studies have found that TSPO may play a role in the regulation of steroidogenesis by its involvement in the translocation of cholesterol to the inner mitochondrial membrane (69). Indeed, one study assessed acute effects of midazolam on adult mice LC steroidogenesis and reported a dose-dependent increase in T production within 1–48 h (28). The stimulatory effect was accompanied by increased TSPO and STAR protein expressions, without changes in the steroidogenic enzymes CYP11A1 and HSD3B, suggesting that activation of TSPO can acutely increase T production. However, the study exposed the cells to very high concentrations (150–600 μM) of midazolam that were two orders above the effective concentrations found in the current study. These results clearly indicate that low-dose repeat exposures of midazolam for an extended period of time may affect LC development by a different mechanism. Further *in vivo* studies are required to confirm the physiological relevance of the current findings.

## Conclusion

We have found that a neurological drug midazolam has the potential to affect LC proliferation and differentiation if given for an extended period of time during the developmental period. The effects were dose-dependent (a stimulating effect at low concentrations and an inhibiting effect at higher concentrations). Increase in T production at lower levels may be due to upregulation in SCARB1 and CYP17A1, while the reductions at higher levels could be due to changes in multiple steroidogenic proteins and/or cofactors. These changes in steroidogenic pathway proteins could be partially mediated by changes in the AKT-CREB signaling pathway. P4 accumulation reflected the concentrations of reductions in T production. In addition to changes in T production, increases in P4 also may impact male reproduction. Since the midazolam concentrations tested (0.1–30 μM) are close to the blood levels found in human patients (0.25–3.5 μM) (42–44), there is a risk that these effects could occur in patients. Future work is needed to evaluate the potential effects on human cells and the mechanisms involved.

## Data Availability Statement

Original contributions presented in the study are included in the article/**Supplementary Material**. Further inquiries can be directed to the corresponding authors.

## Ethics Statement

The animal study was reviewed and approved by Institutional Animal Care and Use Committee of Wenzhou Medical University.

## Author Contributions

XZ, MJ, XW, LY, and HC worked on conception and design. The experiments were performed by XZ, MJ, XW, DC, FH, XG, JT, JX, JS, JW, and LH. XZ, MJ, and XW performed validation and data curation. XZ, MJ, XW, HL, and LY contributed to data curation and project administration. XZ, MJ, and XW provided methodology. XZ, MJ, XW, and HC wrote the paper. LY and HC provided supervision and resources for the study. All authors contributed to the article and approved the submitted version.

## Funding

This work was financially sponsored by National Natural Science Foundation of China (Grant Nos. 91949123, 81771635) and Wenzhou Major Scientific and Technological Innovation Project (Grant No. ZY2019002).

## Conflict of Interest

The authors declare that the research was conducted in the absence of any commercial or financial relationships that could be construed as a potential conflict of interest.

## Publisher’s Note

All claims expressed in this article are solely those of the authors and do not necessarily represent those of their affiliated organizations, or those of the publisher, the editors and the reviewers. Any product that may be evaluated in this article, or claim that may be made by its manufacturer, is not guaranteed or endorsed by the publisher.

## Abbreviations

Acta2, actin alpha 2, smooth muscle actin; Adgre1, adhesion G protein-coupled receptor e1 (Emr1, gene for F4/80); AKT, protein kinase B; ALC, adult Leydig cells; ARX, Aristaless-related homeobox gene; BMP4, bone morphogenetic protein 4; BSA, bovine serum albumin; CD51, cluster of differentiation 51 (integrin alpha V); CD90, cluster of differentiation 90 (Thy-1); CREB, cAMP response element-binding protein; Ct, cycle threshold; CYP11A1, cytochrome P450 family 11A1; CYP17A1, cytochrome P450 family 17A1; DAPI, 4′,6-diamidino-2-phenylindole dihydrochloride; Ddx4, dead-box helicase 4; DHH, desert hedgehog; DIM, differentiation inducing medium; EDS, ethane dimethane sulfonate; EDTA, ethylenediaminetetraacetic acid; EdU, ethynyl-2′-deoxyuridine; EGF, epidermal growth factor; EM, expansion medium; FACS, fluorescence-activated cell sorting; FBS, fetal bovine serum; FGF2, fibroblast growth factor 2; FITC, fluorescein isothiocyanate; Gli1, glioma-associated oncogene homolog 1, zinc finger protein 1; HBSS, Hank’s balanced salt solution; HSD3B, hydroxysteroid dehydrogenase 3b; HSD17B, hydroxysteroid dehydrogenase 17b; IGF-1, insulin-like growth factor; Insl3, insulin-like 3; IS, internal standard; ITS, insulin/transferrin/selenium tissue culture supplement; LC, Leydig cell; LH, luteinizing hormone; LHCGR, luteinizing hormone/choriogonadotropin receptor; LIF, leukemia inhibitory factor; MACS, magnetic-activated cell sorting; NAD, nicotinamide adenine dinucleotide; NADPH, reduced nicotinamide adenine dinucleotide phosphate; NR2F2, nuclear receptor subfamily 2, group F, member 2; P4, progesterone; p75NTR, p75 neurotropin receptor; PBS, phosphate-buffered saline; PDGF, platelet-derived growth factor; PDGFRα, platelet-derived growth factor receptor alpha; PE, phycoerythrin; PI3K, phosphoinositide-3-kinase; Ptprc, protein tyrosine phosphatase receptor type c; Rps16, ribosomal protein S16; SAG, smoothened agonist; SCARB1, scavenger receptor class b member 1; SDS-PAGE, sodium dodecyl sulfate–polyacrylamide gel electrophoresis; SLC, stem Leydig cell; Smc1b, structural maintenance of chromosomes 1b; Sox9, SRY-box transcription factor 9; STAR, steroidogenic acute regulatory protein; T, testosterone; Tcf21, transcription factor 21 (POD-1); TSPO, translocator protein; Vwf, Von Willebrand factor.
