# Supplementary material for: Effects of Midazolam on the Development of Adult Leydig Cells From Stem Cells In Vitro
Source: Front Endocrinol (Lausanne). 2021 Nov 12;12:765251. doi: 10.3389/fendo.2021.765251 (PMC8632869; doi:10.3389/fendo.2021.765251)
Supplement: Supplementary file 1 [file Table_1.docx]

Supplementary Material

# Supplementary Tables

## Supplementary Table S1: Reagents and Chemicals

| **Reagents and Chemicals** | **Manufacturer** | **Catalog number** |
| --- | --- | --- |
| Acetonitrile | Thermo-Fisher | 75058 |
| Anti-R-Phycoerythrin (PE) Magnetic Particles | BD biosciences | 557899 |
| Albumin Bovine V (BSA) | Sigma-Aldrich | A8020 |
| B27 supplement | Thermo-Fisher | 12587010 |
| BD IMag™ Buffer (10X) | BD biosciences | 552362 |
| β-mercaptoethanol | Thermo-Fisher | 21985023 |
| CellTiter 96® Aqueous One Solution | Promega | G3582 |
| Chicken embryo extract (CEE) | Biological | C3999 |
| Click-it® EdU Alexa Fluor Kit | Thermo-Fisher | C10337 |
| Collagenase Type IV | Thermo-Fisher | 9001121 |
| Dexamethasone | Sigma-Aldrich | D4902 |
| DMEM /F12 medium | Sigma-Aldrich | D2906 |
| Enhanced BCA Protein Assay Kit | Beyotime | P0010 |
| Ethane dimethane sulfonate (EDS) | SKS Chem | 4672495 |
| Epidermal growth factor (EGF) | Peprotech | 315-09 |
| Fetal bovine serum (FBS) | Sigma-Aldrich | 10099-141 |
| Fibroblast growth factor (FGF2) | Thermo-Fisher | RP-8626 |
| Insulin-transferrin-sodium selenitemedia supplement (ITS) | Sigma-Aldrich | I1844 |
| Leukemia Inhibitory Factor (LIF) | Millipore | L5158 |
| Luteinizing hormone (LH) | MyBiosource | MBS313432 |
| LightCycler® 480 SYBR® Green I Master | Roche | 04887352001 |
| Methanol | Thermo-Fisher | 67561 |
| Midazolam | Jiangsu Enhua | H19990027 |
| N2 supplement | Thermo-Fisher | 17502048 |
| Non-essential amino acids (NEAA) | Thermo-Fisher | 11140050 |
| Oncostatin-M | Prospec | CYT-169 |
| Platelet-derived growth factor (PDGF-BB) | Prospec | CYT-412 |
| Progesterone solution | Sigma-Aldrich | P069 |
| Reverse Transcription System | Promega | A3500 |
| RIPA Lysis Buffer | Beyotime | P0013C |
| RNeasy® Mini Kit | Qiagen | 74104 |
| Smoothened agonist (SAG) | Cayman Chemical | 11914 |
| Testosterone solution | Sigma-Aldrich | T070 |
| Testosterone-d3 solution | Sigma-Aldrich | T046 |
| Trion X-100 | Solarbio | T8200 |
| Trypsin-EDTA | Thermo-Fisher | 25200072 |
| 24-well plate round cell slipper | WHB | WHB24CS |

## Supplementary Table S2: Manufacturer and Dilutions of Antibodies

| **Antigen** | **Antibody** | **Dilution** | **Manufacturer (Cat#)** |
| --- | --- | --- | --- |
| α -Tubulin | Rabbit pAb | 1:1000 | Beyotime Biotech (AF0001) |
| AKT | Rabbit mAb | 1:1000 | Cell Signaling Technology (9272) |
| PE-CD90 | Mouse monoclonal | 1:100 | Thermo-Fisher (12-0900-83) |
| CREB | Rabbit mAb | 1:1000 | Cell Signaling Technology (9197) |
| CYP11A1 | Rabbit mAb | 1:1000 | Cell Signaling Technology (14217) |
| CYP17A1 | Rabbit monoclonal | 1:1000 | Abcam (ab125022) |
| HSD3B1 | Mouse mAb | 1:500 | Novus (NB110-78644) |
| INSL3 | Rabbit monoclonal | 1:500 | Abcam (ab65981) |
| LHCGR | Rabbit pAb | 1:500 | MULTI SCIENCES (ab7496-050) |
| pAKT | Rabbit mAb | 1:1000 | Abcam (ab192623) |
| pCREB | Rabbit mAb | 1:500 | Abcam (ab32096) |
| SCARB1 | Rabbit monoclonal | 1:1000 | Abcam (ab52629) |
| STAR | Rabbit mAb | 1:500 | Cell Signaling Technology (8449) |
| Anti-Mouse | Goat IgG (H+L) HRP | 1:3000 | MULTI SCIENCES (GAM007) |
| Anti-Rabbit | Goat IgG (H+L) HRP | 1:3000 | MULTI SCIENCES (GAR007) |
| Anti-Rabbit | Goat Dylight 488 | 1:1000 | MULTI SCIENCES (GAR4882) |
| Anti-Rabbit | Goat Dylight 594 | 1:1000 | MULTI SCIENCES (GAR5492) |

## Supplementary Table S3: QPCR Primer Sequences

| **Genes** | **Forward (5'to3')** | **Reverse (5'to3')** |
| --- | --- | --- |
| Acta2 | GGCTATGCTCTGCCTCAT | GGACGATCTCACGCTCA |
| Adgre1 | GCAACCTGCCACAACAC | GAGGGACCACAAAGCGT |
| Cd90 | GCACCATGAACCCAGTCA | CGGCAGTCCAGTCGAAG |
| Cyp11a1 | CCCTGCTGGAAGGTGTAG | AGGCAAAGCGGAATAGGT |
| Cyp17a1 | TCATTACACCCACGCAGA | GCGGGGCAGTTGTTTAT |
| Ddx4 | GCATTCCCATTGTGTTAGC | TGGCAGTTATTCCATCCC |
| Gli1 | CCCAAGTTTCTGGGAGGTT | TGAAGGGGCAGGATAGGA |
| Hsd3b1 | CAGTGTATGTAGGCAATGTG | AAGGCAAGCCAGTAGAGC |
| Insl3 | AGGGCTCAGGTGGTGTGT | TGTGGTCCTTGGTTTCTGG |
| Lhcgr | CCCCTTATCACTGTCACCA | CGTTTACAGCAGCCGAAT |
| Pdgfr-a | TCCCAGGCTTTGGCTCT | GGGGTATCTCGGCTTCCT |
| Ptprc | CAAGACAGAAGGGTGCAGA | TGGTGTTTAGATGCTGGCT |
| Rps16 | CGATAAGCCAGTCTCAAGG | TCACTCCAGTCCCCACA |
| Smc1b | AAGACCAAGAAGGCAGCA | CCACAGGCTCAGGGTAAA |
| Sox9 | TCTCCTAACGCCATCTTCA | TGCACGTCTGTTTTGGG |
| Star | CATCCAGCAAGGAGAGGAAG | CACCTGGCACCACCTTACTT |
| Vwf | GGGCCTTTCTGTGGTCTG | GGGTCTTCCTCCACCTGA |

## Supplementary Table S4: Protein Molecular Weights of Western Blots.

| **Protein** | **Manufacturer** | **Molecular weight ( kDa)** |
| --- | --- | --- |
| α -Tubulin | Beyotime Biotech | 55 |
| AKT | Cell Signaling Technology | 60 |
| CREB | Cell Signaling Technology | 43 |
| CYP11A1 | Cell Signaling Technology | 50 |
| CYP17A1 | Abcam | 55 |
| HSD3B1 | Novus | 42 |
| INSL3 | Abcam | 14 |
| LHCGR | MULTI SCIENCES | 79 |
| pAKT | Abcam | 56 |
| pCREB | Abcam | 37 |
| SCARB1 | Abcam | 80 |
| STAR | Cell Signaling Technology | 28 |
